# Supplementary material for: Spatiotemporal mapping of major trauma in Victoria, Australia
Source: PLoS One. 2022 Jul 6;17(7):e0266521. doi: 10.1371/journal.pone.0266521 (PMC9258853; doi:10.1371/journal.pone.0266521)
Supplement: S1 File — (PDF) [file pone.0266521.s001.pdf]

SUPPLEMENTARY MATERIAL

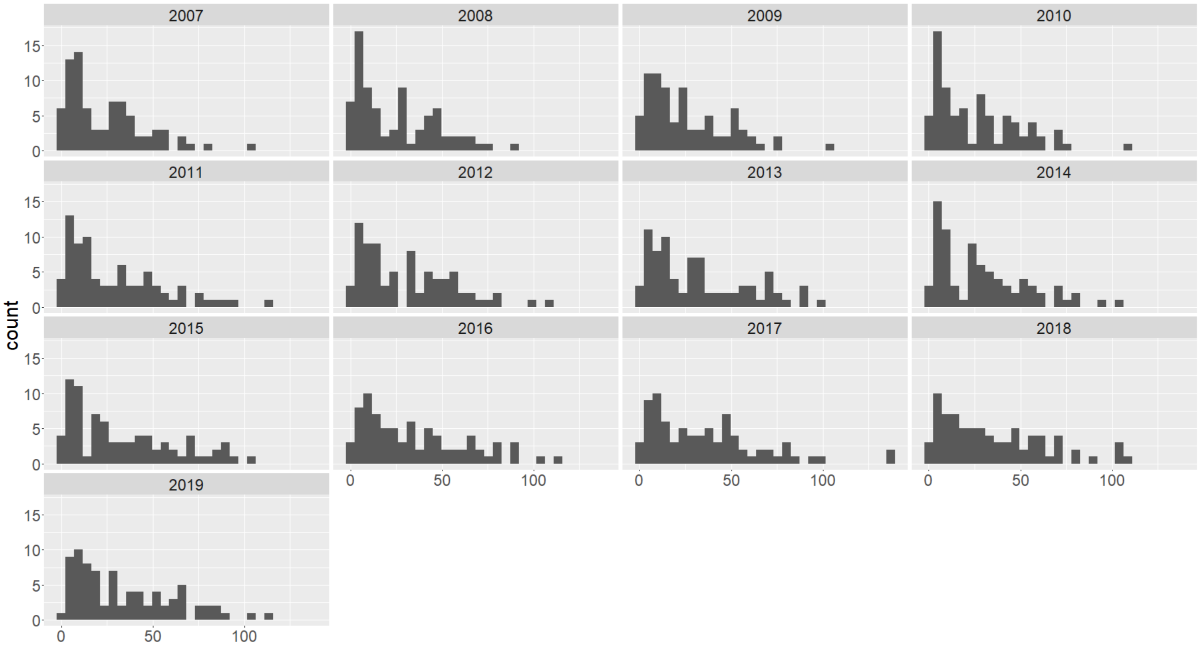

Figure 1: Histogram of the number of major trauma cases per LGA per year.

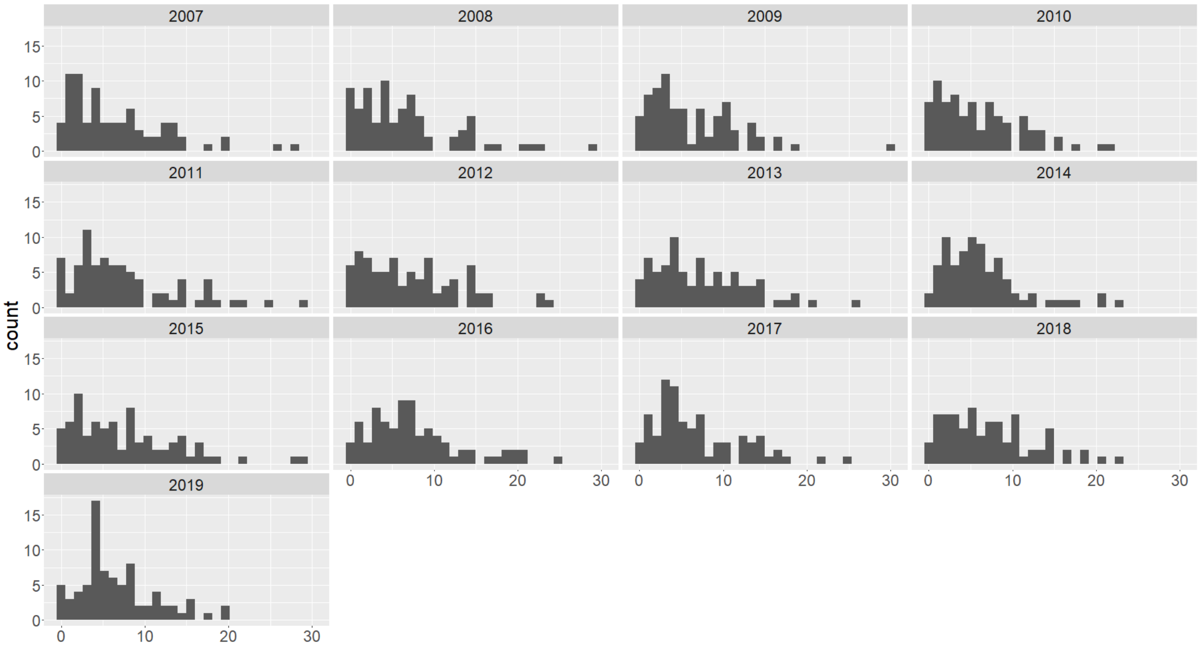

Figure 2: Histogram of the number of motor-vehicle collision cases per LGA per year.

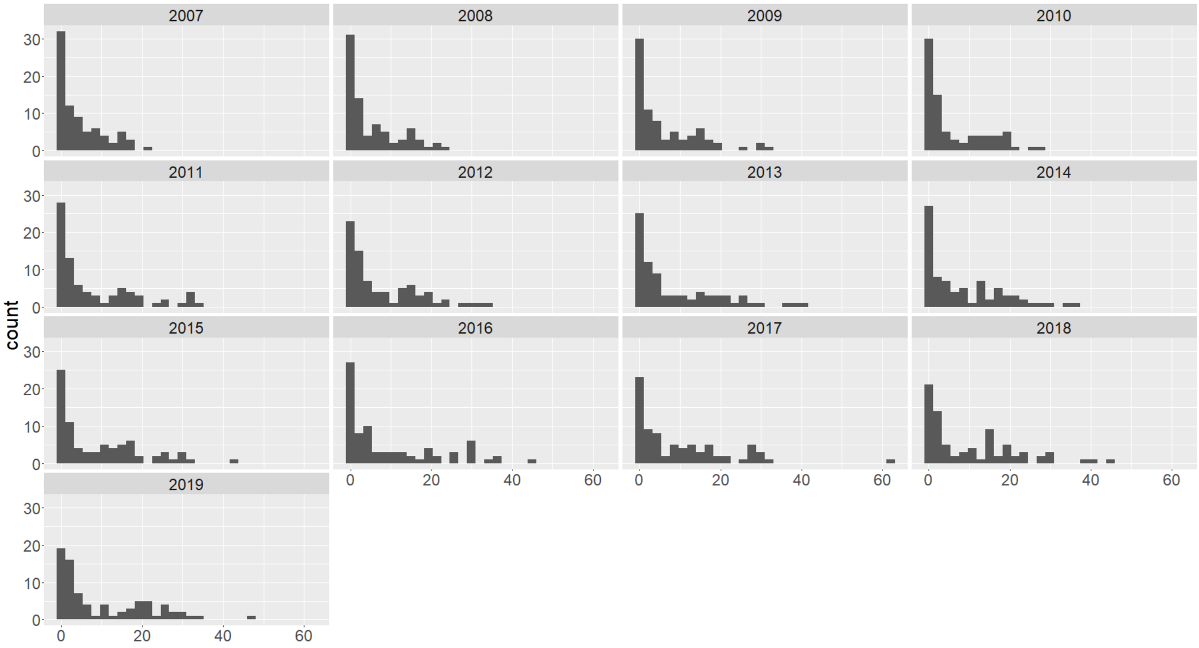

Figure 3: Histogram of the number of low fall cases per LGA per year.

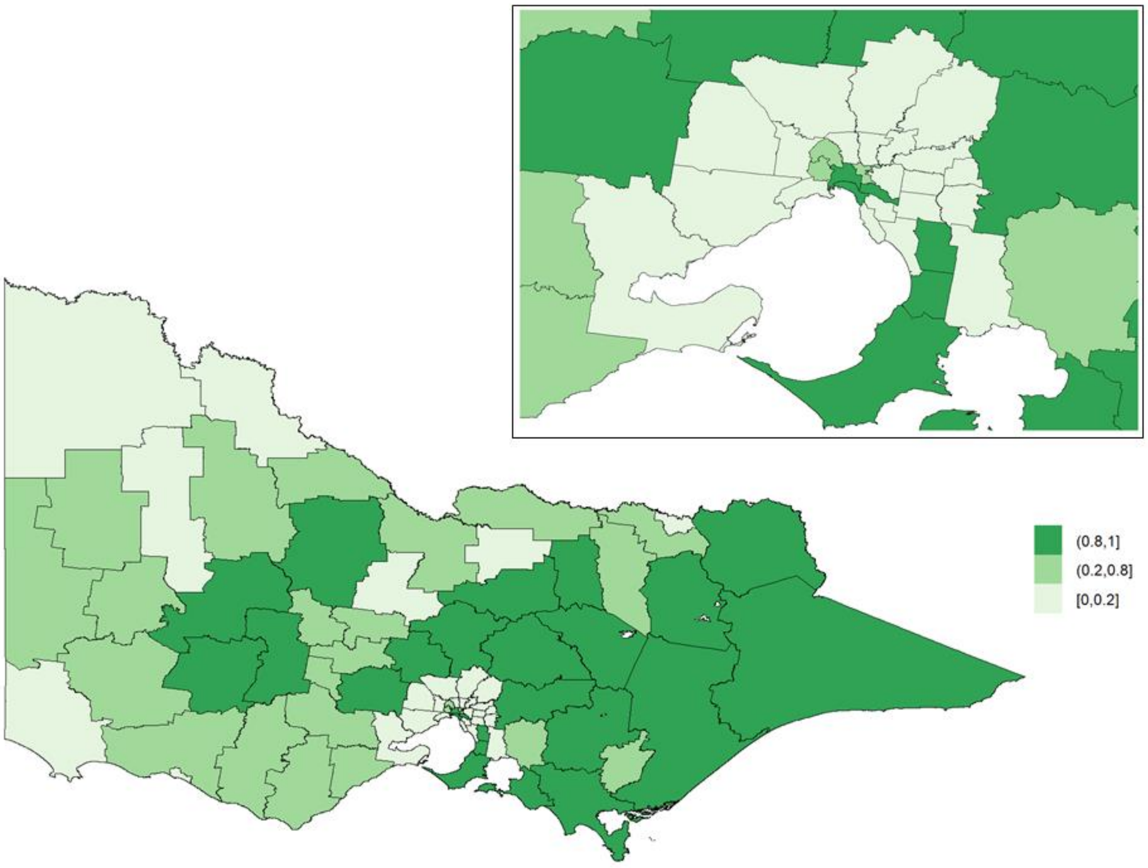

Figure 4: Posterior probability of the area-specific spatial risk being greater than 1 for major trauma overall. The inset on the top-right shows Greater Melbourne.

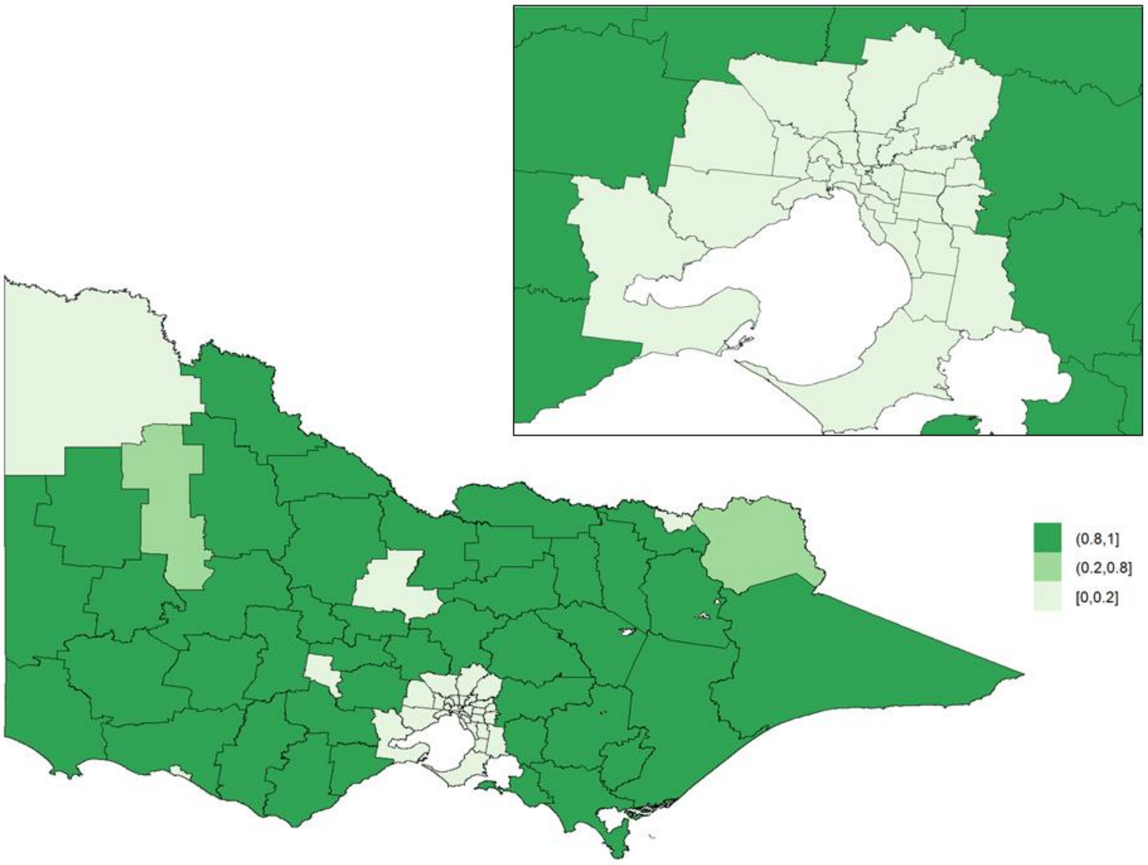

Figure 5: Posterior probability of the area-specific spatial risk being greater than 1 for injury by motor vehicle collision. The inset on the top-right shows Greater Melbourne.

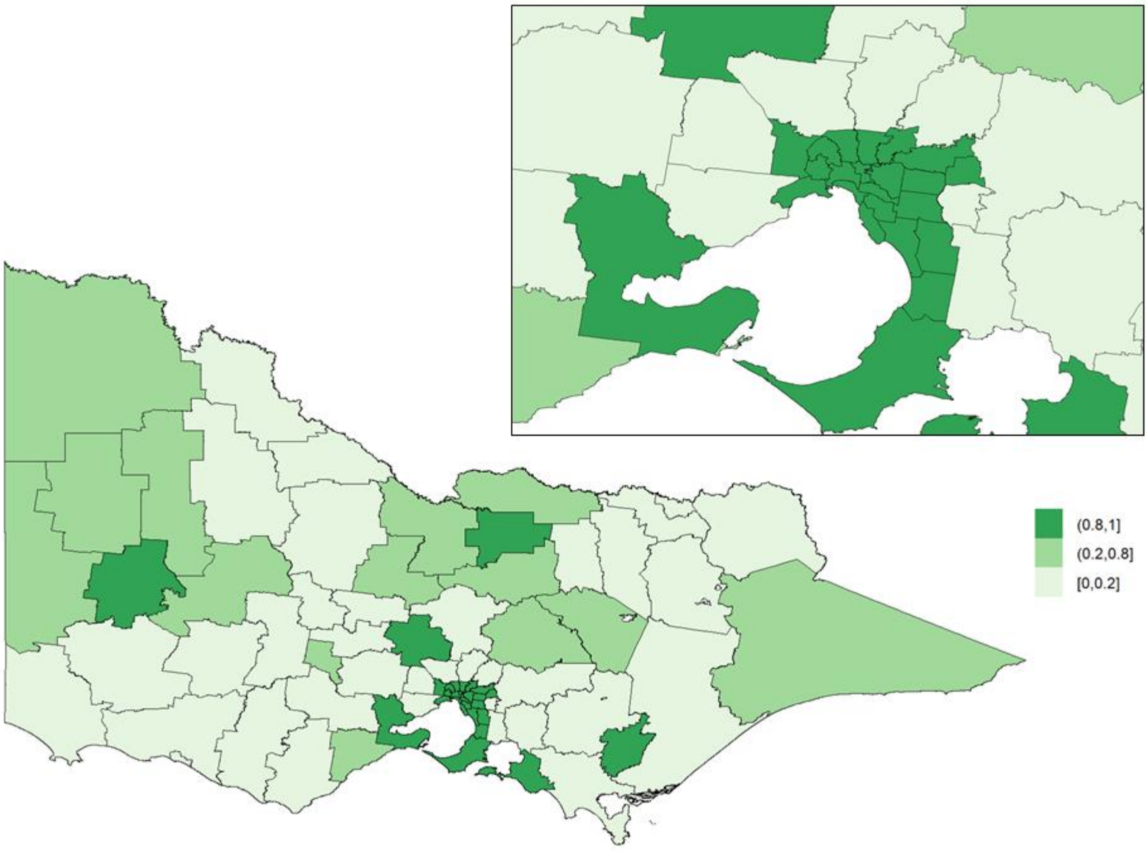

Figure 6: Posterior probability of the area-specific spatial risk being greater than 1 for injury by low fall. The inset on the top-right shows Greater Melbourne.

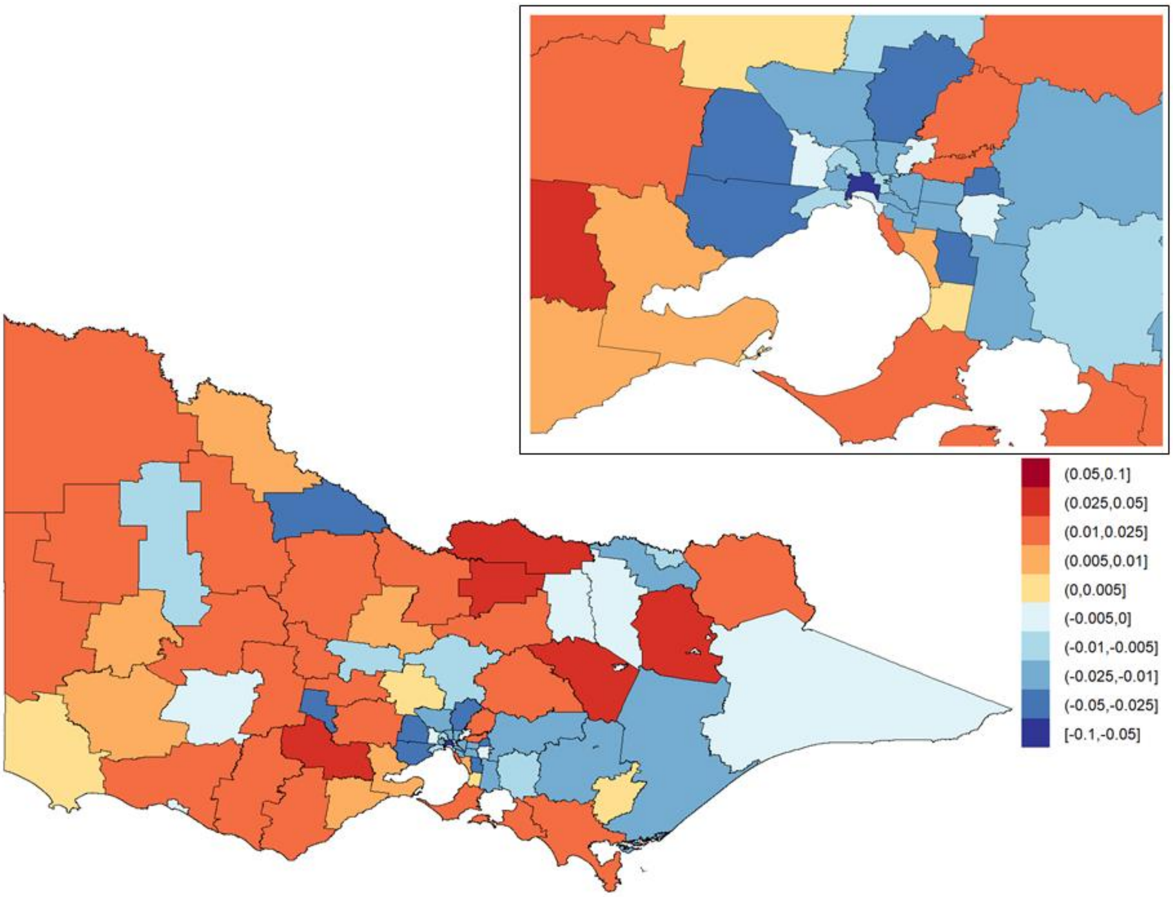

Figure 7: Differential time trend (on the linear scale) for major trauma overall in Victoria (with an inset map of Greater Melbourne). Here, a value of 0 indicates that the area-specific risk at any given year is the same as that in the whole of Victoria in that year; a value of greater than 0 indicates that the area-specific risk is increasing more year-on-year than in Victoria overall; and a value of less than 0 indicates that the area-specific risk is increasing less year-on-year than in Victoria overall.

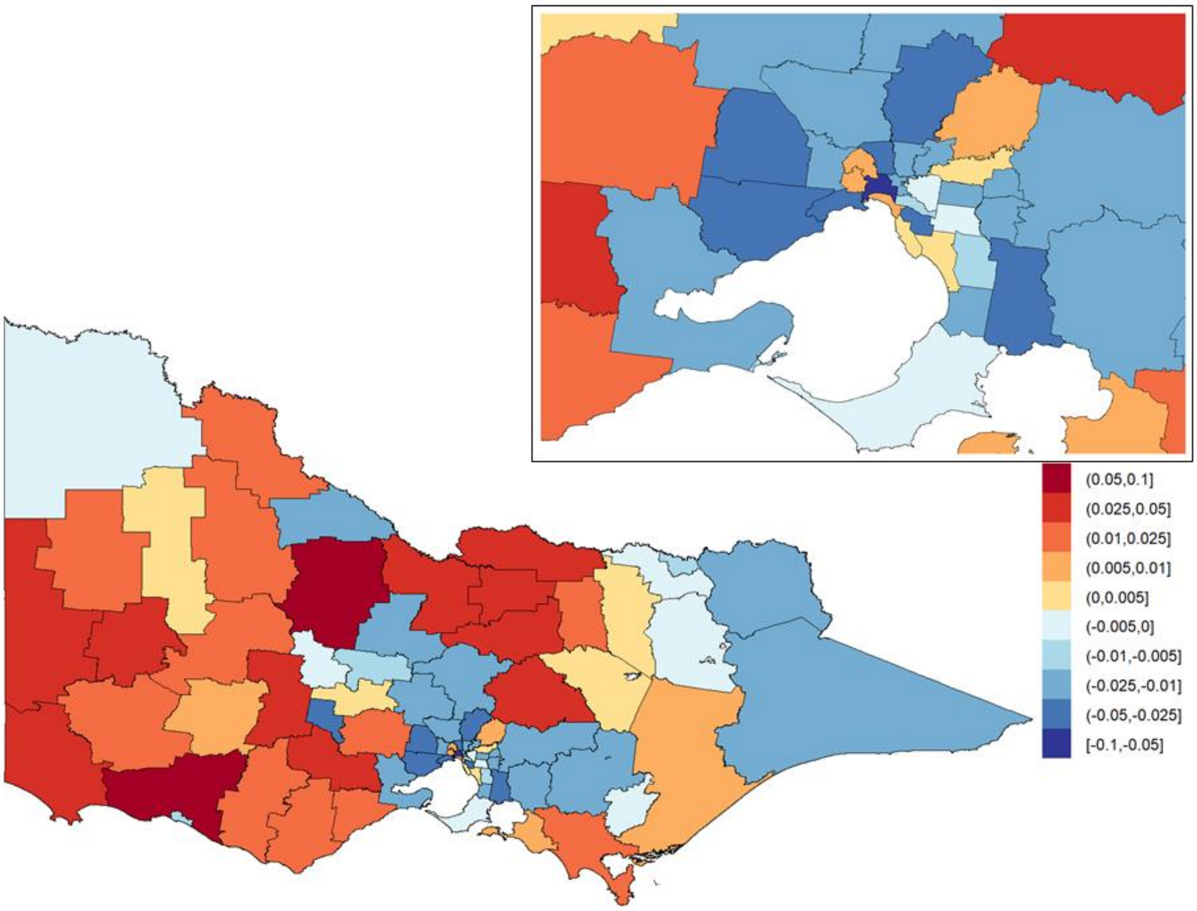

Figure 8: Differential time trend (on the linear scale) for injury by motor vehicle collision in Victoria (with an inset map of Greater Melbourne). Here, a value of 0 indicates that the area-specific risk at any given year is the same as that in the whole of Victoria in that year; a value of greater than 0 indicates that the area-specific risk is increasing more year-on-year than in Victoria overall; and a value of less than 0 indicates that the area-specific risk is increasing less year-on-year than in Victoria overall.

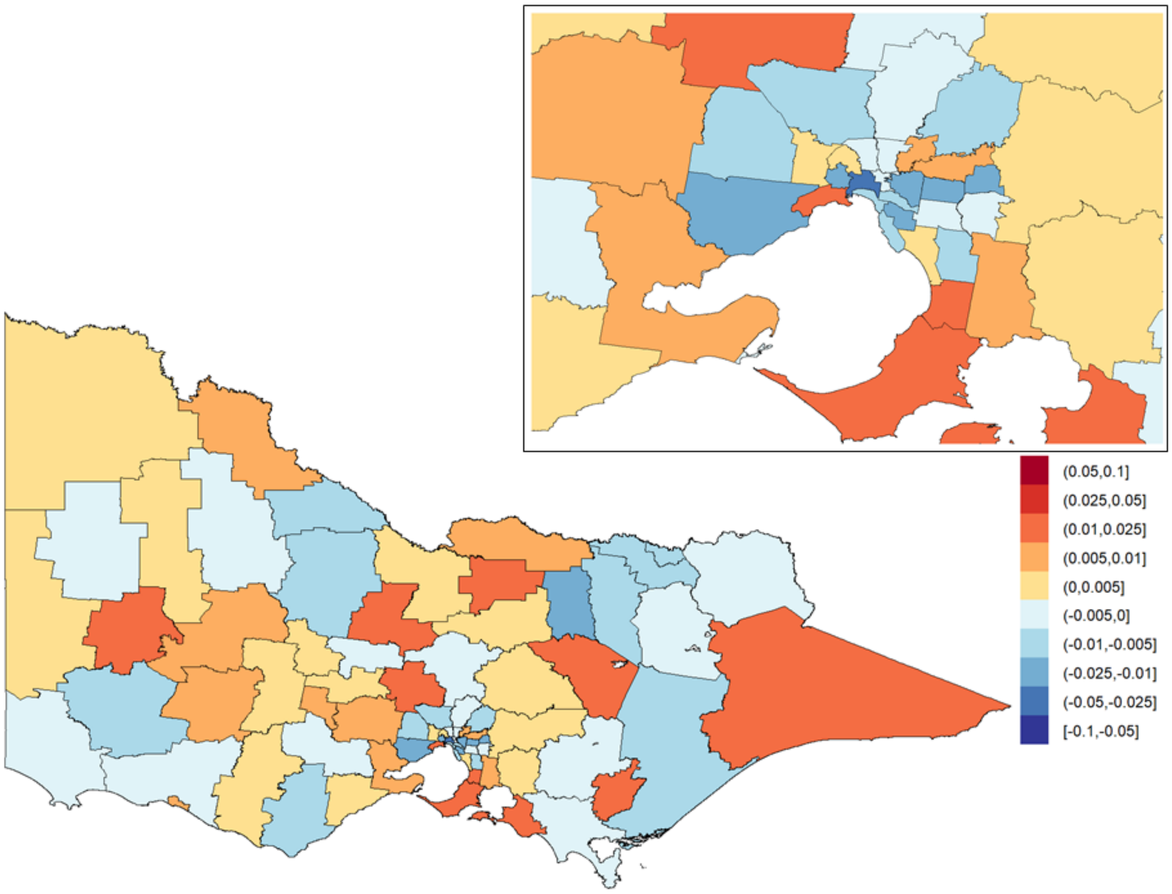

Figure 9: Differential time trend (on the linear scale) for injury by low fall in Victoria (with an inset map of Greater Melbourne). Here, a value of 0 indicates that the area-specific risk at any given year is the same as that in the whole of Victoria in that year; a value of greater than 0 indicates that the area-specific risk is increasing more year-on-year than in Victoria overall; and a value of less than 0 indicates that the area-specific risk is increasing less year-on-year than in Victoria overall.

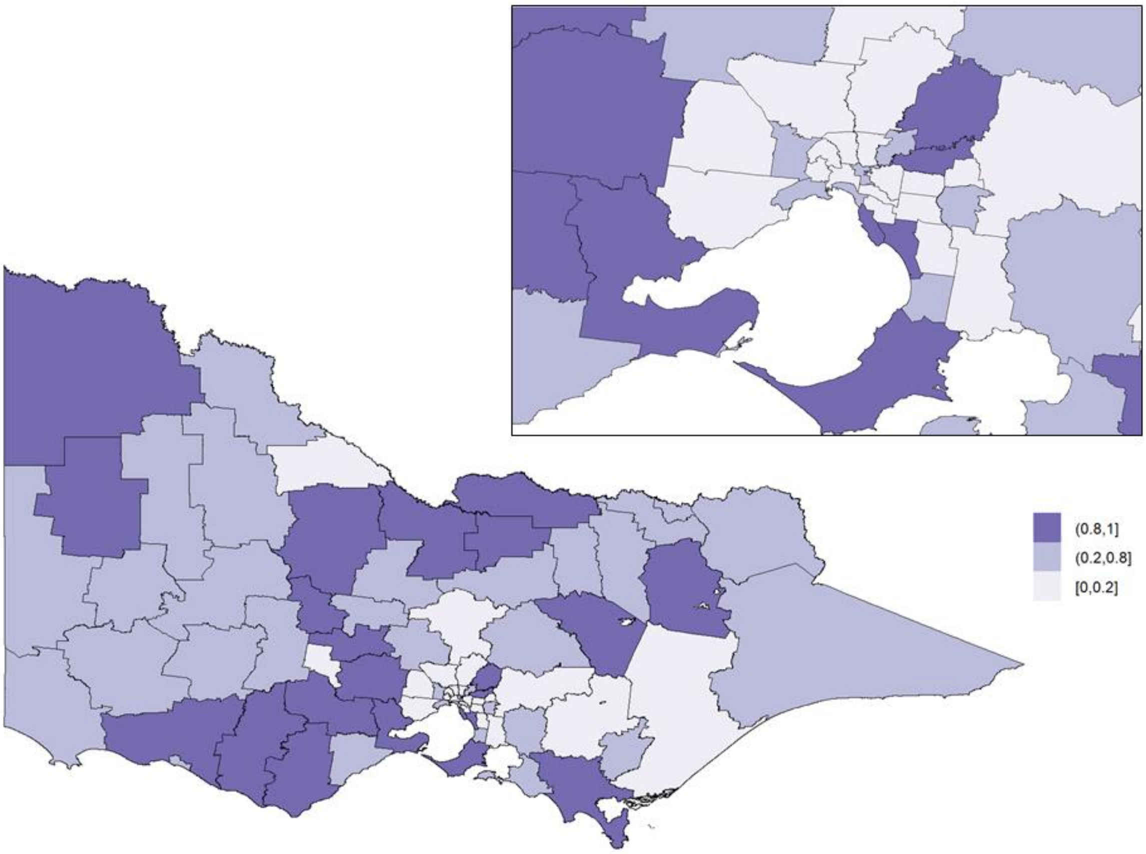

Figure 10: Posterior probability of the area-specific yearly multiplicative change in risk being greater than 1 for major trauma overall. The inset on the top-right shows Greater Melbourne.

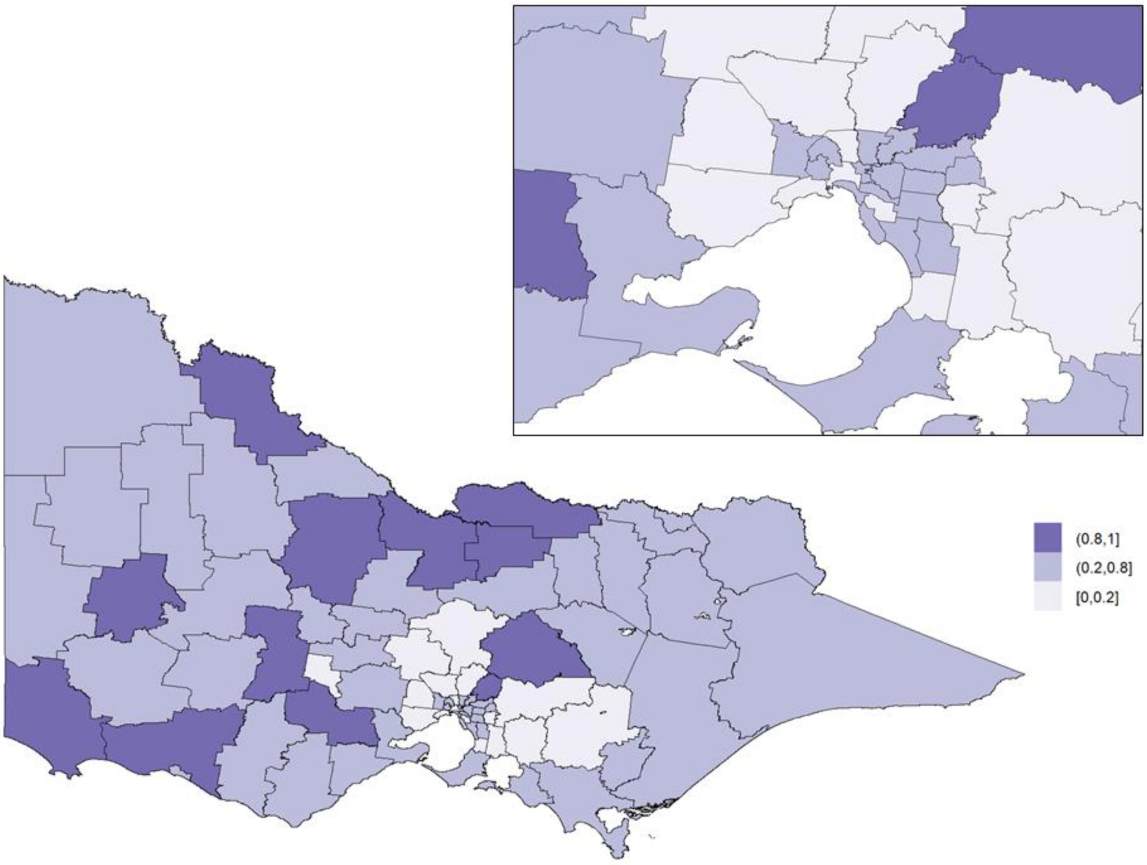

Figure 11: Posterior probability of the area-specific yearly multiplicative change in risk being greater than 1 for injury by motor vehicle collision. The inset on the top-right shows Greater Melbourne.

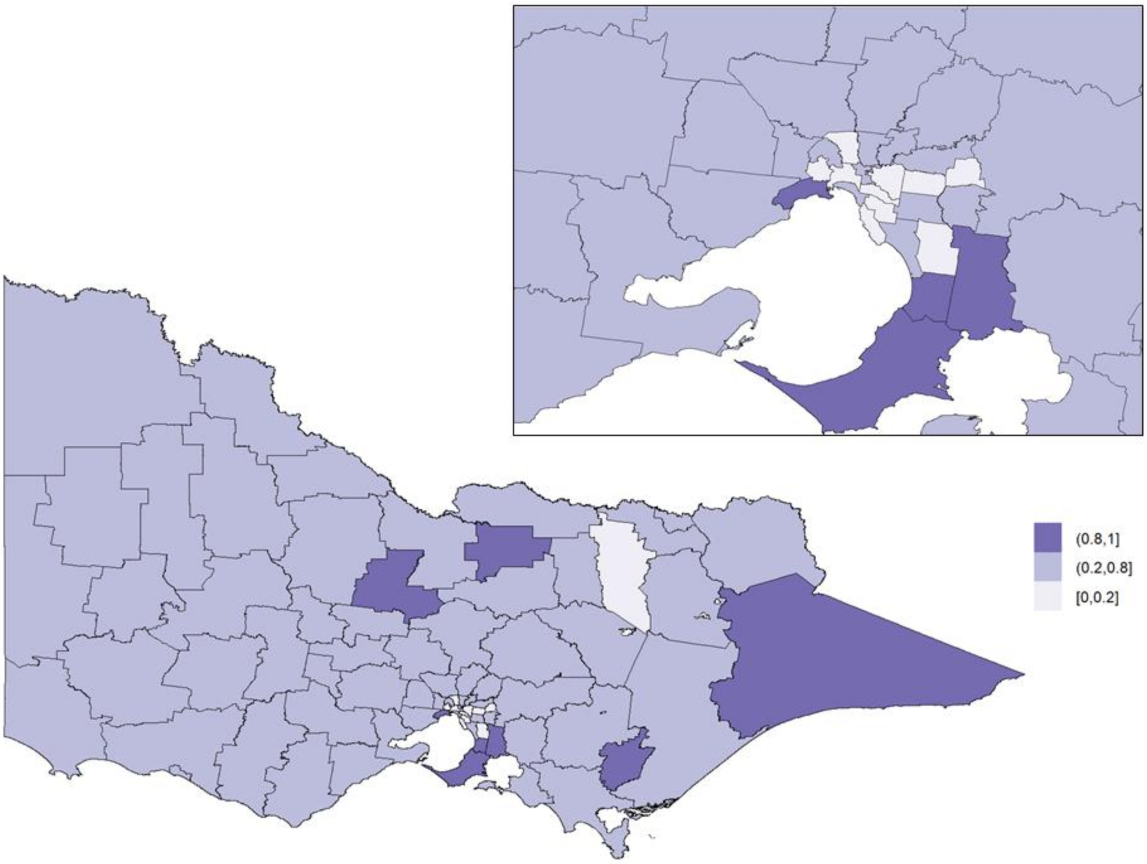

Figure 12: Posterior probability of the area-specific yearly multiplicative change in risk being greater than 1 for injury by low fall. The inset on the top-right shows Greater Melbourne.
